# The Institutional Worker

**Published:** 1911-12-02

**Authors:** 


THE
### INSTITUTIONAL WORKER
Being a Special Supplement to "The Hospital"

### Editor's Notices.

**Contributions:**

Contributions should be written, or preferably typed, on one side of the paper only, and all articles sent in are accepted upon the distinct understanding that they are forwarded to The Hospital only.

The Editor cannot undertake to return MSS. not used, but when a stamped directed envelope is enclosed the MSS. may be returned if a special request is made.

Accepted articles and paragraphs of news will be paid for after publication at the scale rate.

**Address:**

To prevent delay all contributions and letters on editorial business must be addressed exclusively to the Editor, "The Hospital," 29 Southampton Street, Strand, London, W.C. It is important that this regulation shall be strictly observed.

**Correspondence:**

Correspondence on all subjects is invited. The name and address of correspondents must be given as a guarantee of good faith, but not necessarily for publication.

**Special Articles:**

Special articles are invited, and questions, inquiries, and paragraphs upon all matters relating to the work, administration, and management of general, special, mental, fever, cottage and convalescent hospitals, sanatoria, homes, institutions, societies and organisations for the treatment or care of the sick, injured and dependents of all classes. Every matter affecting the interests and welfare of the staff of all grades working in these institutions will receive special consideration.

**Administrative Medicine, Research and Items of News:**
Special terms will be paid for approved articles on subjects in this wide field by experts who have made some section of it their special study and interest. We wish to give prominence to every movement tending to advance accuracy of diagnosis, efficiency in treatment, and the development of modern methods to eradicate disease and promote the welfare of the suffering.

**A Bureau of Information:**
We invite inquiries and applications for information and help in providing for that numerous class of sufferers who require special care or house-room which they cannot obtain in their own homes or secure for themselves. **We cannot, however, prescribe or recommend practitioners.**

**Books for Review:**
Publishers are particularly requested to send advance proofs of any new books of importance whenever possible, as the Editor has made arrangements to publish immediate reviews on a new plan.

**Photographs, Plans, Blocks, and Illustrations:**
It is requested that wherever possible MSS. may be accompanied by illustrations in any of the above forms. The name of the sender and of the article to which it belongs should be written on the back of each photograph, drawing, or block for purposes of identification.

**Local Papers:**
Newspapers containing reports or news paragraphs **should be marked** and addressed to the Sub-Editor.

**The Coupon System:**
A coupon will be found at the bottom of the third inside page of the cover each week. This coupon must be attached to every question or inquiry to which an answer is desired in The Hospital.

### Manager's Notices.

Letters relating to the publication, sale, and advertising departments of The Hospital must be addressed to the Manager, "The Hospital," The Hospital Building, 28 and 29 Southampton Street, Strand, London, W.C.

**Subscriptions** which may begin at any time, are payable in advance. Cheques and Post-Office orders, crossed London County and Westminster Bank, Covent Garden branch, should be made payable to the Manager of The Hospital and addressed to him at The Hospital Building, 28 and 29 Southampton Street, Strand, London, W.C.

**Advertisements:**

All advertisements for The Hospital must reach the Manager not later than Wednesday morning in each week. For scale of charges see pages v and 2.

**Classified Advertisements:**

All small advertisements will be classified, for this section of the paper is widely read and of special interest. We wish to encourage those wanting appointments who are attracted to institutional work, and therefore offer them the reduced rate of *twenty-one words and under for* 1s., and *for every ten and part of ten words beyond,* 6*d.*

### POINTS FOR INSTITUTION AUTHORITIES.

The economical management of an Institution depends very greatly upon purchasing supplies in the cheapest market.

It is often impossible locally to secure the highest quality at the lowest prices.

In consequence, a section will be reserved in this part of the paper for announcements of Institutions inviting Tenders for Contracts.

This will enable Authorities of Institutions to secure tenders for supplies from all over the country, and ensure purchases at the lowest possible prices consistent with quality.

The charge for Tenders is 6s. per inch, and for this small outlay many pounds may be saved on supplies of all descriptions.

**Special Rates** will be quoted for those institutions which agree to send all their vacancy, contract, and public notices to The Hospital each year, so as to make it a file of such announcements for ready reference.

### POINTS FOR TRADE ADVERTISERS.

The Hospital is the organ of Institutions, whether of a Voluntary Character or those under Local Government, Poor Law and Municipal Authorities.

It is read by all Executive Officials and those responsible for the Construction, Management, and Equipment of Public Establishments.

It is also the Journal of the worker engaged in the Medical, Health and Preventive services.

There is no medium more appropriate and effective in the promotion of business in these directions than The Hospital.

### POINTS FOR INSTITUTIONAL WORKERS.

A very large number of Appointments Vacant appear every week in the columns of The Hospital.

If you cannot find a position suited to your requirements, make your wishes known through our Situations Wanted Column.

### APPOINTMENTS VACANT.

### Poor-Law Vacancies.

### Appointments Vacant.

ARCHITECTURAL ASSISTANT (£2 weekly), Bolton Union. Mr. H. I. Cooper, C. to G., 28 Mawdsley Street, Bolton. Dec. 2.

ASSISTANT ATTENDANT AND SCHOOL TEACHER (£25), Prescot Union. Mr. A. F. Mann, C. to G., Whiston, Prescot. Dec. 4.

ASSISTANT FEMALE RELIEVING OFFICER (£75), Norwich Guardians. Mr. E. J. W. Huggins, C. to G., Norwich.

ASSISTANT MASTER AND STOREKEEPER (£50), Brentford Union. Mr. W. Stephens, C. to G., Isleworth, W. Dec. 5.

ASSISTANT MATRON (£25), Hartley Wintney Union. Mr. W. H. Wright, C. to G., Odiham, Hants.

ASSISTANT NURSE (£25), Pontefract Union. Mr. T. J. Amies, C. to G., Union Offices, Pontefract. Dec. 8.

ASSISTANT NURSE (£22), Bermondsey Guardians. E. Pitts Fenton, C. to G., 283 Tooley Street, S.E. Dec. 11.

ASSISTANT NURSE (£31), West Ham Union. T. Smith, C. to G., Union Road, Leytonstone, N.E. Dec. 6.

ASSISTANT NURSE (£28), Eastry Union. Mr. F. S. Cloke, C. to G., Sandwich. Dec. 4.

ASSISTANT NURSE (£20), King's Lynn Union. Mr. R. C. Coulton, C. to G., Lynn.

ASSISTANT RATING SURVEYOR (£150), Bolton Union. Mr. H. I. Cooper, C. to G., 28 Mawdsley Street, Bolton. Dec. 2.

BOYS' INDUSTRIAL TRAINER (£20), Salisbury Union. Mr. E. Mould, C. to G., Salisbury. Dec. 7.

CHARGE DAY NURSE (£30), Guildford Union. W. S. V. Cullerne, C. to G., Commercial Road, Guildford. Dec. 8.

CHARGE NURSE (£30), Haslingden Union. J. H. Sinkinson, C. to G., Union Offices, Rawtenstall. Dec. 12.

CHARGE NURSE (£30), West Ham Union. T. Smith, C. to G., Union Road, Leytonstone, N.E. Dec. 6.

CHARGE NURSE (£30), South Shields Union. J. W. Coulson, C. to G., Barrington Street, S. Shields. Dec. 16.

CHARGE NURSES (£30), Pontypridd Union. Mr. W. Spickett, C. to G., Pontypridd. Dec. 8.

CHARGE NURSES (£24), City of London Union. Matron, City of London Infirmary, Clifden Road, N.E.

CHILDREN'S ATTENDANT (£20), Wandsworth Union. Matron, Intermediate Schools, Swaffield Road, Wandsworth.

CLASS SISTER (£50), West Ham Union. T. Smith, C. to G., Union Road, Leytonstone, N.E. Dec. 6.

DRILL MISTRESS (£35), St. Pancras Guardians. Mr. J. E. P. Hall, C. to G., Town Hall Pancras Road, N.W. Dec. 5.

FEMALE ATTENDANT (£20), Bermondsey Guardians. Matron of the Workhouse, Ladywell, S.E.

FEMALE INDUSTRIAL TRAINER (£24), Louth Union. Mr. F. C. Chard, C. to G., Louth. Dec. 2.

FEMALE SICK ATTENDANTS (£28), Edmonton Union. Mr. F. Shelton, C. to G., Lower Tottenham, N. Dec. 4.

FIRST-CLASS FEMALE ATTENDANT (£25), Lambeth Guardians. Matron of the Parish School, Elder Road, West Norwood, S.E.

FOSTER-MOTHER (£24), Atcham Union. Mr. E. P. Everest, C. to G., Shrewsbury. Dec. 2.

FOSTER-MOTHER (£22), Aston Union. Mr. J. North, C. to G., Vauxhall Road, Birmingham. Dec. 4.

FOSTER-MOTHER (£30), Aylesbury Union. Mr. F. H. Parrott, C. to G., Aylesbury. Dec. 7.

GENERAL FEMALE RELIEF ATTENDANT (£25), West Ham Union. Mr. T. Smith, C. to G., Leytonstone, N.E. Dec. 6.

INFANTS' HELPER (£16), Hackney Union. Matron of the Branch Workhouse, Brentwood.

INFANTS' NURSE (£30), Bridgend and Cowbridge Union. Mr. R. H. Cox, C. to G., Bridgend, Glam. Dec. 2.

JUNIOR ASSISTANT NURSES (£15), Bishop's Stortford Union. A. G. Gwynn, C. to G., Bishop's Stortford. Dec. 9.

LABOUR MASTER (£40), Kidderminster Union. Mr. F. E. Burcher, C. to G., Kidderminster. Dec. 9.

LABOUR MASTER (£30), Hartley Wintney Union. Mr. W. H. Wright, C. to G., Odiham, Hants.

LABOUR MISTRESS (£25), Sheffield Union. Mr. A. E. Booker, C. to G., Sheffield. Dec. 2.

LUNATIC ATTENDANT (£30), St. Pancras Guardians. Mr. J. E. P. Hall, C. to G., Town Hall, Pancras Road, N.W.

MALE LUNATIC ATTENDANT (£30), City of London Union. Medical Officer, 42 Clifden Road, Lower Clapton, N.E.

MALE NIGHT NURSE (27s. weekly, non-resident). Mr. A. J. Harris, C. to G., Cardiff. Dec. 11.

MALE NURSE (23s. weekly), West Ham Union. Mr. T. Smith, C. to G., Leytonstone, N.E. Dec. 6.

MALE NURSE (£30), Chelsea Guardians. Mr. C. W. Shepherd, C. to G., 250 King's Road, Chelsea, S.W. Dec. 4.

Male Nurse (£25), King's Lynn Union. Mr. R. C. Coulton, C. to G., Lynn.

Male Receiving Ward Attendant (£28), Brighton Guardians. Mr. B. Burfield, C. to G., Brighton. Dec. 5.

Master and Matron (£80 and £50), Carlisle Union. Mr. H. B. Lonsdale, C. to G., Carlisle. Dec. 11.

Master's Assistant Clerk (£25), Bermondsey Guardians. Mr. E. Pitts Fenton, C. to G., 283 Tooley Street, S.E. Dec. 7.

Nurse (£30), Pontefract Union. Mr. T. J. Amies, C. to G., Union Offices, Pontefract. Dec. 8.

Nurse (£30), Falmouth Union. Mr. R. N. Rogers, C. to G., Falmouth. Dec. 6.

Nurse (£30), Fylde Union. F. H. Browne, C. to G., Union Offices, Wesham, Kirkham. Dec. 12.

Nurse (£25), Hartley Wintney Union. W. H. Wright, C. to G., Odiham, Hants.

Nurse (£30), Ticehurst Union. J. Chase Edwards, C. to G., Union Offices, Wadhurst, Sussex. Dec. 16.

Nurse (£30), West Derby Union. Mr. H. P. Cleaver, C. to G., Brougham Terrace, Liverpool. Dec. 7.

Nurse (£27 10s.), Stockbridge Union. Matron of the Workhouse, Stockbridge. Dec. 4.

Nurse (25), Worksop Union. Mr. J. S. Whall, C. to G., Worksop. Dec. 5.

Nurse (£30), Stanford Union. Mr. R. M. English, C. to G., Stanford.

Nurses (£25), Lewes Union. C. Patrick, C. to G., Union Offices, Lewes. Dec. 7.

Nursery Attendant (£20), Islington Guardians. Matron of the Workhouse, St. John's Road, Upper Holloway, N.

Probationers (£10), King's Norton Union. Mr. R. J. Curtis, C. to G., Guildhall Buildings, Birmingham.

Probationers (£6), St. Pancras Guardians. J. E. P. Hall, C. to G., Town Hall, Pancras Road, N.W.

Sister (£35), West Derby Union. Mr. H. P. Cleaver, C. to G., Brougham Terrace, Liverpool. Dec. 7.

Staff Nurses (£26), Sheffield Union. Mr. A. E. Booker, C. to G., Westbar, Sheffield. Dec. 7.

Superintendent Nurse (£35), Poole Union. Mr. P. E. L. Budge, C. to G., Poole. Dec. 5.

Temporary Staff Nurses (3) (£20), Holborn Union. Matron of the Infirmary, Archway Road, Upper Holloway, N.

Ward Sister (£30), Bermondsey Guardians. E. Pitts Fenton, C. to G., 283 Tooley Street, S.E. Dec. 6.

Ward Sister (£30), Hammersmith Guardians. C. to G., 206 Goldhawk Road, Shepherd's Bush. Dec. 11.

### Situations Vacant.

Assistant Cook (£35), Edmonton Union. Matron of the Infirmary, Bridport Road, Upper Edmonton, N.

Assistant Laundress (£24), Bath Union. Mr. W. E. Winckworth, C. to G., Bath. Dec. 9.

Attendant and Ironer (£20), Halstead Union. Mr. R. L. Hughes, C. to G., Halstead.

Barber and General Assistant (£25), Long Ashton Union. Mr. A. E. Hicks, C. to G., Flax Bourton. Dec. 4.

Cook-General for Superintendent's House (£20), Hackney Union. Matron of the Children's Homes, Chipping Ongar.

Head Cook (£45), Edmonton Union. Matron of the Infirmary, Bridport Road, Upper Edmonton, N.

Laundress (£27), Chelmsford Union. Mr. A. S. Duffield, C. to G., 96 High Street, Chelmsford. Dec. 6.

Laundress (£22 10s.), Boston Union. Mr. J. M. Simpson, C. to G., Boston. Dec. 7.

Laundress (£27 10s.), Thetford Union. Mr. J. Houchen, C. to G., Thetford. Dec. 7.

Male Bath Attendant (£30), Bermondsey Guardians. Mr. E. Pitts Fenton, C. to G., 283 Tooley Street, S.E. Dec. 4.

Porter (£25), Swindon and Highworth Union. Mr. J. P. Kirby, C. to G., Swindon. Dec. 2.

Porter (£28), King's Lynn Union. Mr. R. C. Coulton, C. to G., King's Lynn. Dec. 12.

Porter, Etc. (£25), Gainsborough Union. Mr. D. M. Robbs, C. to G., Gainsborough. Dec. 2.

Temporary Cook (30s. weekly), Edmonton Union. Matron of the Infirmary, Bridport Road, Upper Edmonton, N.

Wardwomen (£18), West London District Schools. Matron of the Schools, Ashford, Middlesex.

### Institutional and Administrative Vacancies.

### Appointments Vacant.

Matron (£150), Anglo-American Hospital, Cairo, Egypt. Secretary. Dec. 20.

Matron (£40), Stratton Cottage Hospital, N. Cornwall. Hon. Sec., Sanctuary, Stratton. Dec. 6.

Staff Nurse, Birmingham and Midland Skin Hospital. Apply Matron.

### Municipal Vacancies.

### Appointments Vacant.

Advisory Water Engineer, Farsley U.D.C. Particulars from Clerk to Council.

Architectural Draughtsman (42s. weekly), Margate T.C. Mr. E. Brooke, Town Clerk, Margate. Dec. 4.

Assistant Sanitary Inspector (£80), Witney R.D.C. Mr. N. J. G. Ravenor, Clerk to the Council, 22 High Street, Witney, Oxon. Dec. 2.

Assistant Superintendent of Sewage Pumping Station (£156), West Ham T.C. Mr. F. E. Hilleary, Town Clerk, West Ham. Dec. 2.

Charge Nurse (£30), Hebburn Urban District Council Isolation Hospital. T. Stuart, Clerk to Council. Dec. 2.

Clerk (Engineer's Dept.) (£150), Hampstead B.C. Town Clerk. Dec. 4.

Clerk (Town Clerk's Office) (£104), Portsmouth T.C. Town Clerk. Dec. 7.

Divisional Road Surveyors (4) (£220 each), Southampton C.C. Mr. H. Barber, Clerk to the Council, Winchester. Dec. 4.

Engineer and Manager for Gas Works (£200), Cleckheaton U.D.C. Mr. J. H. Linfield, Clerk to the Council, Cleckheaton. Dec. 6.

Health Visitor (£75), Great Yarmouth Town Council. W. E. Stephens, Town Clerk. Dec. 12.

Health Visitor (£100), Metropolitan Borough of Poplar. L. Potts, Town Clerk, High Street, Poplar. Dec. 4.

Junior Clerks (£70 minimum), M.A.B. Mr. T. Duncombe Mann, Clerk to the Board, Embankment, E.C. Dec. 5.

Lady Health Visitor (£75), City of Wakefield. Mr. W. W. Greenhalgh, Town Clerk. Dec. 12.

Land Agent (£250), Lancashire C.C. Mr. H. E. Clare, Clerk to the Council, Preston. Dec. 2.

Matron (£60), Florence Nightingale Hospital, Bury. The Clerk, District Joint Hospital Board, Cross Street, Bury, Lancs. Dec. 12.

Nurse (£32 10s.), Lanchester Joint Hospital Board Infectious Hospital. W. H. Ritson, Clerk, Lanchester, Durham. Dec. 4.

Surveyor and Inspector of Nuisances (£150), Seaham Harbour U.D.C. Mr. H. B. Wright, Clerk to the Council, Seaham Harbour. Dec. 5.

Surveyor of Highways (£120), Holsworthy R.D.C. Mr. C. Kinsman, Clerk to the Council, Holsworthy. Dec. 5.

Temporary Nurse (£24), Croydon Rural and Merton Joint Hospital Board. Dr. C. M. Fegen, Council Offices, Katharine Street, Croydon.

### Situations Vacant.

Cook (£28-30), Coventry City Hospital. Apply Matron. Dec. 6.

Cook-General (£20), Cheadle R.D.C. Isolation Hospital. Mr. F. S. Cox. Clerk, Union Offices, Cheadle, Staffs Dec. 5.

